# Saliva exposure reduces gingival keratinocyte growth on TiO_2_-coated titanium

**DOI:** 10.1007/s10856-024-06792-0

**Published:** 2024-04-18

**Authors:** Sini Riivari, Nagat Areid, Elisa Närvä, Jaana Willberg, Timo Närhi

**Affiliations:** 1 Department of Prosthetic Dentistry and Stomatognathic Physiology, University of Turku, FI-20520 Turku, Finland; 2 Institute of Biomedicine and Cancer Research Laboratory FICAN West, University of Turku, FI-20520 Turku, Finland; 3 Department of Oral Pathology and Oral Radiology, University of Turku, FI-20520 Turku, Finland; 4 Turku University Hospital and University of Turku, FI-20520 Turku, Finland; 5 Wellbeing Services County of South-West Finland and University of Turku, FI-20520 Turku, Finland

## Abstract

Bioactive, nanoporous TiO_2_-coating has been shown to enhance cell attachment on titanium implant surface. The aim of this study was to evaluate, whether the saliva proteins affect the epithelial cell adhesion on TiO_2_-coated and non-coated titanium. Grade V titanium discs were polished. Half of the discs were provided with TiO_2_-coating produced in sol with polycondensation method. Half of the TiO_2_-coated and non-coated discs were treated with pasteurized saliva for 30 min. After saliva treatment, the total protein amounts on surfaces were measured. Next, the hydrophilicity of discs were measured with water contact angle measurements. Further, the gingival keratinocyte adhesion strength was measured after 2 and 6 h of cultivation using serial trypsinization. In addition, cell growth and proliferation were measured after 1, 3, and 7 days of cell culture. Finally, cell morphology, spreading and adhesion protein signals were detected with high resolution confocal microscopy. As a result, in sol coated TiO_2_-surface had significantly higher hydrophilicity when compared to non-coated titanium, meanwhile both non-coated and TiO_2_-coated surfaces with saliva treatment had a significant increase in hydrophilicity. Importantly, the amounts of adhered saliva proteins were equal between TiO_2_-coated and non-coated surfaces. Adhesion strength against enzymatic detachment was weakest on non-coated titanium after saliva exposure. Cell proliferation and cell spreading were highest on TiO_2_-coated titanium, but saliva exposure significantly decreased cell proliferation and spreading on TiO_2_-coated surface. To conclude, even though saliva exposure makes titanium surfaces more hydrophilic, it seems to neutralize the bioactive TiO_2_-coating and decrease cell attachment to TiO_2_-coated surface.

**Graphical Abstract:**

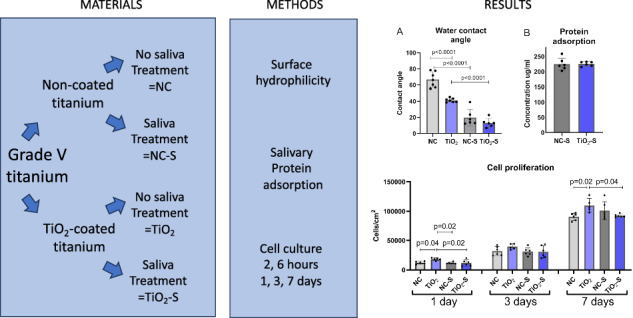

## Introduction

Dental implant materials have been developed to achieve appropriate biocompatibility. Important material properties that affect cell and tissue integration to implant surface are for example surface roughness, nanotexture, chemistry and surface wettability [1, 2]. Nevertheless, in oral conditions dental materials are in contact with saliva most of the time, and saliva exposure can modify earlier mentioned surface properties as saliva is able to produce a thin film on biomaterials [3]. Saliva consists mostly of water, but it also contains different salivary proteins, minerals, enzymes and serum albumin [4, 5]. Saliva includes over thousand different proteins; proline-rich proteins, statherins, cystatins, histatins, amylase and mucins to name the major families [6]. These salivary proteins are able to adhere to dental material surfaces and can change the surface properties. Salivary proteins bind preferably on surfaces with high roughness values [7].

Peri-implantitis is a biofilm-associated disease, which occurs, when oral microbes invade to peri-implant area, causing an inflammation in peri-implant mucosa and leading to initiation of peri-implant bone resorption [8, 9]. There are many factors that expose to peri-implantitis. Poor oral hygiene, a history of periodontitis, smoking and diabetes have been often reported [10]. One crucial factor behind peri-implantitis is a weaker soft tissue barrier around the implant abutment than around the natural tooth. The gingival fibres in connective tissue are not able to attach directly to the implant surface, rather they form a capsule-like structure, which allows an easier access for oral bacteria into deeper peri-implant tissue [11, 12]. However, the epithelium is able to attach to implant surface in a similar manner to the natural tooth, via hemidesmosomes and basal lamina [13]. Hemidesmosomes’ main function is cell adhesion by binding the cytoplasmic plaque to basal lamina. In addition, the hemidesmosomes take part in cell signalling [14]. Important molecules in basal lamina are laminins, of which the laminin-332 plays the most important role concerning gingival epithelial cell adhesion [15]. Laminins can bind to cell membrane-penetrating integrins and thus affect cell spreading, growth and migration. Laminin-332 binds specifically to integrin α6β4 [16–19], which again is able to bind intracellular adapter proteins forming a connection between the intracellular cytoskeleton and extracellular basal lamina [20].

Nanoporous, bioactive TiO_2_ -coatings have been shown to have favourable effects on soft tissue cell attachment to titanium and zirconia surfaces [21–23]. The benefits of sol gel-derived TiO_2_-coatings comprise, that they are thin, hydrophilic, bioactive, nonresorbable and rather easy to produce [24]. The dip-coating sol-gel method which has been used in many previous studies has limitations when coating objects with variable surface shapes. This study uses, in sol-produced TiO_2_-coating which is based on polycondensation and facilitates coatings on a wider selection of implant components. In addition, it allows faster coating procedures in normal laboratory circumstances without the need for special equipment. Moreover, this coating has shown to produce nanotopography on titanium surfaces and increase cell spreading and adhesion on abutment surface in vitro [25, 26].

However, even though cell response to TiO_2_ surface has been shown to be favorable in vitro, it ought to be noted, that as the implant crown or abutment is connected, at least the coronal part will be in contact with saliva. This can cause significant changes in bioactive surface properties and thus affect the cell and tissue adherence [1]. The aim of this study was to determine, whether there is a difference in surface properties after saliva exposure between nanoporous TiO_2_-coated titanium and non-coated titanium surface and does the saliva exposure affect gingival keratinocyte attachment on TiO_2_-coated and non-coated titanium.

## Materials and methods

### Sample preparation and TiO_2_-coating procedure

The study consisted of four groups: non-coated titanium (NC), non-coated titanium treated with saliva (NC-S), TiO_2_-coated titanium (TiO_2_) and TiO_2_-coated titanium treated with saliva (TiO_2_-S). Factory-made round titanium discs with 10 mm diameter were used (Grade 5, titanium 90%, vanadium 6%, aluminium 4%). The titanium surfaces were polished with rotating 1200 grit sandpaper (LaboPol 21, Struers A/S, Rodovre, Denmark). The grindings were eliminated with 5 min of washing with acetone and ethanol.

Half of the discs were coated with sol gel-derived TiO_2_-coating made directly in sol with polycondensation technique as described earlier by Riivari et al. [25, 26]. To produce the sol, two solutions were prepared. Solution 1 consisted of 28.4 g of titanium isopropoxide (98%, Acros Organics) mixed with 21,2 g of ethanol (95%). Solution 2 was mixed from 4,5 g of 2-ethoxyethanol (99%, Acros Organics), 1,8 g of hydrogen chloride (HCl, 1 M) and 16,7 g of ethanol. Solution 2 was pipetted into the solution 1 while mixing effectively. The produced sol had a transparent colour and was left to age at 0 °C for 24 h. While waiting for the coating procedure, the sol was kept at −18 °C.

The polished titanium discs were coated with a layer of TiO_2_-sol and set in a freezer for two hours (–18 °C). Thereafter, the discs were washed twice with ethanol, placed in a ceramic bowl and heated in an oven until 500 °C, where the discs were kept for 10 min. Further, the acetone and ethanol washing was replied for 5 min each and the discs were sterilized in an autoclave.

### Saliva coating

Paraffin wax stimulated whole saliva was collected from 7 healthy non-smoking adult volunteers for 10 min. The bacteria were eliminated from saliva with pasteurization. First, the saliva was centrifuged (9500 rpm, 40 min) followed by pasteurization at 60 °C degrees for 30 min. After this, the solution was centrifuged again and divided into smaller portions. After pasteurization, the solutions were tested and no microbial growth was detected.

The titanium discs were covered with 1 ml saliva diluted in PBS (1:1) and shaken for 30 min which followed by washing three times with PBS.

### Protein adsorption

After saliva exposure, the amounts of adsorbed saliva proteins on coated and noncoated surfaces were detected. 100 µl of warmed SDS buffer (2%, 95 C) was added to the titanium discs (*n* = 3) and incubated for 5 min. The detachment of proteins was prompted with brushing, all the solution was collected in Eppendorf tubes, boiled for 7 min and centrifuged for 2 min. The solutions were diluted with PBS (1:20) and 150 µl were pipetted into 96-plate with 150 µl of Micro BCA™ Protein Assay Kit (Thermo Scientific™) following 2 h incubation (+37 C). The total protein amounts were measured with wavelength of 562 nm with Multiskan FC reader (Thermo Scientific) and compared the given values to standard curve.

### Water contact angles measurements

The surface hydrophilicity of TiO_2_-coated and non-coated titanium with and without saliva exposure were measured with water contact angle measurements using the sessile drop method (Attension Theta, Biolin Scientific). Altogether, six drops of distilled water for each group were used at room temperature (*n* = 6 technical replicates). Each drop was imaged for 10 s after dropping and the mean contact angle value was determined.

### Cell cultures

Spontaneously immortalized human gingival keratinocytes (hGKs) obtained from gingival biopsy by Mäkelä et al. [27] with the passage of 20 were used as a cell type. The hGKs were mixed in keratinocyte-serum-free medium (SFM) (Gibco®, Thermo Fisher, USA).

#### Cell adhesion strength against enzymatic detachment

To measure the adhesion strength against enzymatic detachment, the hGKs were cultured at a density of 12,500 cells/cm^2^ on NC, NC-S, TiO_2_ and TiO_2_-S surfaces for 2 and 6 h (*n* = 6/group/time point). Attachment strength was measured with serial trypsinization earlier described by Meretoja et al. [22] After 2 and 6 h of cell attachment, the discs were washed with PBS and set on trypsin solution (0.005% trypsin (Gibco, Invitrogen) diluted in PBS (1:5)). The discs were incubated at room temperature for 20 min replacing trypsin after 1, 5 and 10 min collecting the solution to cryotubes. After, the discs were treated with undiluted trypsin at 37 °C for 5 min to detect the number of adherent cells. To all tubes, 500 µl of TE-Triton X-100 was added and frozen at -70 °C. Afterward, the amount of released DNA was measured with PicoGreen dsDNA-kit (Molecular Probes Europe, Netherlands). The fluorescence values were detected with wavelengths of 490 and 535 nm. The percentage of detached cells was calculated by comparing the amounts of detached cells to amounts of adherent cells.

#### Cell attachment and proliferation

Long-term cell attachment and growth were studied by cultivating hGKs on titanium discs for 1, 3 and 7 days (*n* = 6/group/time point), which followed treatment with Alamar Blue (Thermo Fischer, USA) blended in SFM. The Alamar Blue solution was incubated on the discs for 3 h in a CO_2_ -incubator at 37 °C degrees. Thereafter, 200 µl from each specimen was used to measure the absorbance of the solution with a wavelength of 569 and 594 nm (Multiskan FC, Thermo Scientific). The cell amounts were calculated by comparing the absorbance values to the standard curve.

### Cell spreading and hemidesmosomes formation

After one day of cell culture, the discs were fixed with paraformaldehyde (4%) for 15 min, washed once with PBS and stored at 4 °C. Later, the discs were treated with 300 µl TRITON-X-100 in PBS (0,5%, 15 min). The primary antibodies [laminin y2 (1:100, sc-7652, Santa-Cruz Biotechnology), integrin *β*4 (1:100, ab182120, Abcam)] were mixed with horse serum in PBS (30%) and the discs were covered with antibody dilution overnight. The next day, the discs were washed three times with PBS and covered with secondary antibody dilution [Anti-Rabbit 488, Anti-Goat 555, (both from ThermoFisher Scientific)], DAPI (nucleus staining, 1:200) and Phalloidin Atto (1:400, Sigma-Aldrich) in 30% horse serum in PBS] for one hour. After staining, the discs were washed in PBS and glued to microscope glass using Mowiol (Sigma-Aldrich). A spinning disc confocal microscope (63x Zeiss Plan-Apochromat, 3i CSU-W1 Spinning Disk) was used to image the stained discs. Cell area was measured from 30 cells from each group using ImageJ Fiji program.

### Data analyses

The data analysis was made with GraphPad Prism-program. One-way analyses of variance (ANOVA) with Tukey’s multiple comparisons test in case of normal distribution and otherwise Kruskal Wallis test were used to analyse the significance of differences. Confocal images were analysed with ImageJ, Fiji-program.

## Results

### TiO_2_ coating and saliva exposure increases hydrophilicity

**Fig. 1:**
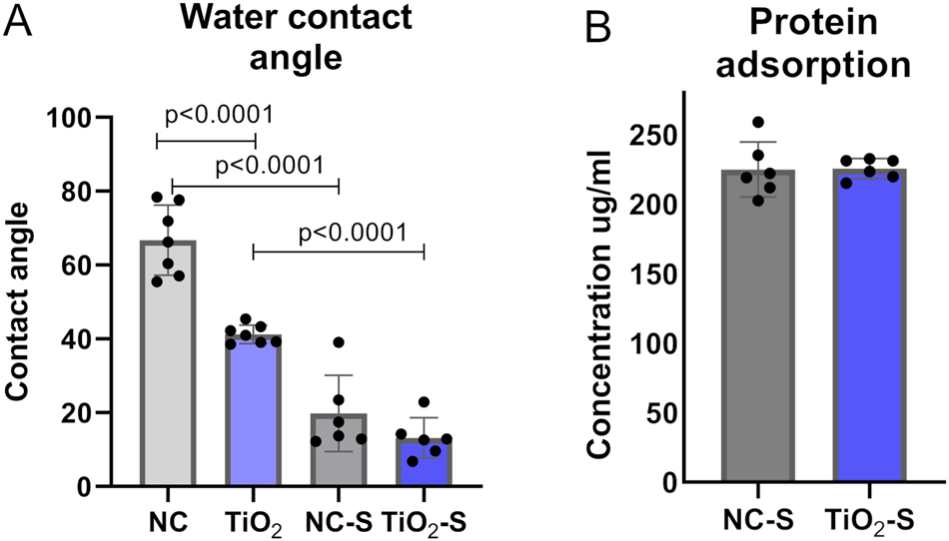
Saliva proteins adsorb equally and increase hydrophilicity of the surfaces (**A**) the water contact angle measurements and (**B**) salivary protein adsorption on non-coated and TiO_2_-coated. NC non-coated, TiO_2_ TiO_2_-coated, NC-S non-coated with saliva exposure, TiO_2_-S=coated with saliva exposure. Mean ± SD+ technical replicates, ANOVA

Whether the TiO_2_-coating and adhered saliva proteins would affect the surface hydrophilicity, the water contact angle measurements were accomplished. TiO_2_-coated surface had significantly lower contact angle values when compared to non-coated titanium. In addition, both non-coated and TiO_2_-coated surfaces with saliva exposure had a significant decrease in contact angles. These results indicate that saliva exposure increase the hydrophilicity of both surface but the effect is more intense on non-coated titanium (Fig. 1A).

### Total saliva protein adsorption is equal between TiO_2_-coated and non-coated titanium

To determine, whether there is a difference in saliva protein adherence on TiO_2_-coated and non-coated titanium, the total protein amount was tested after 30 min of saliva treatment. The amounts of adhered saliva proteins were equal between TiO_2_-coated and non-coated surfaces, indicating that the observed difference is not a result of variance between the saliva protein adherence (Fig. 1B).

### Saliva exposure reduces cell adhesion strength on non-coated surfaces

**Fig. 2:**
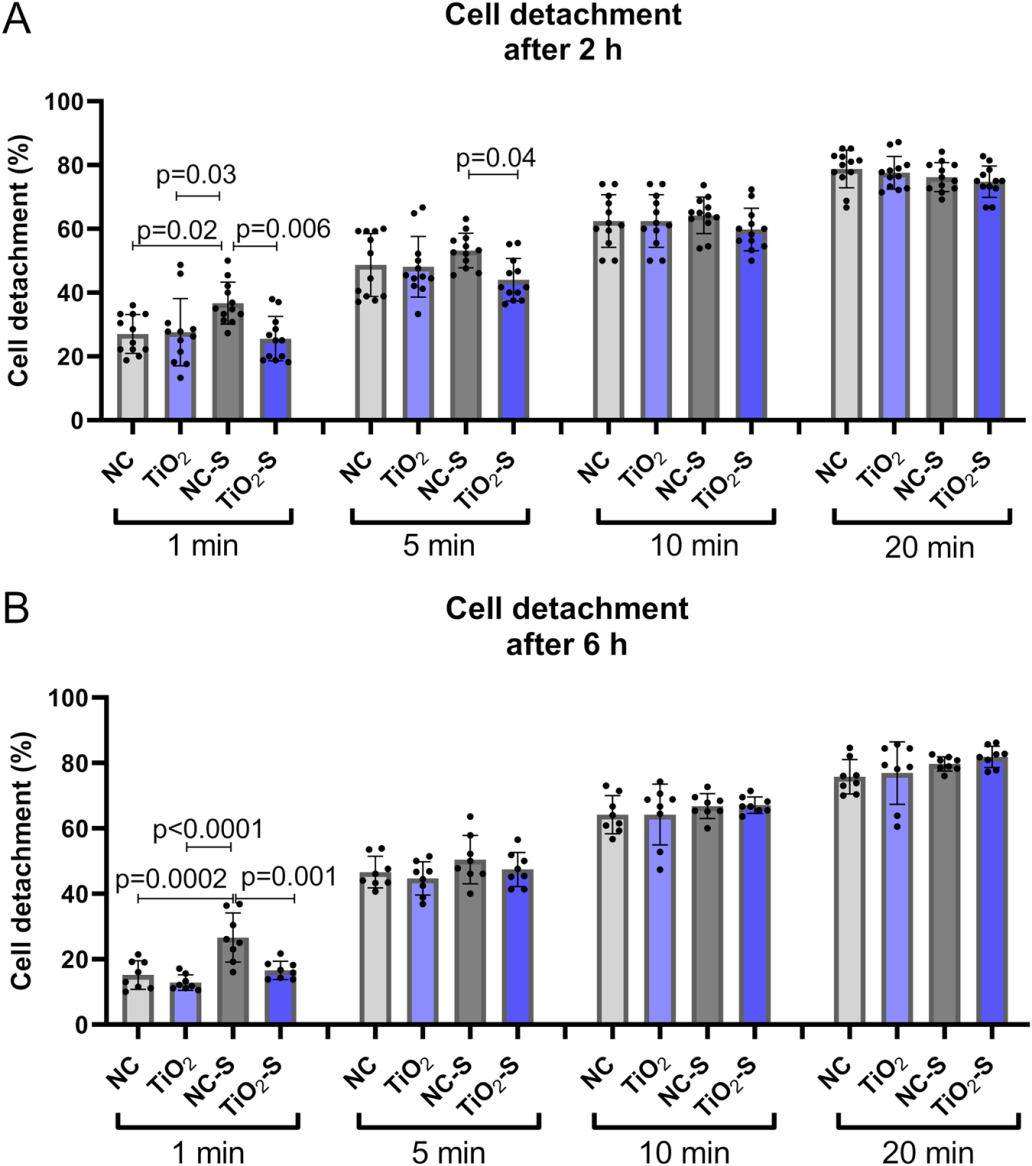
Saliva exposure reduces cell adhesion strength on non-coated surfaces. Cumulative amounts of detached keratinocytes after (**A**) 2 and (**B**) 6 h of cell culture using a method of serial trypsinization after 1, 5, 10 and 20 min of. NC non-coated, TiO_2_ = TiO_2_-coated, NC-S non-coated with saliva exposure, TiO_2_-S = TiO_2_-coated titanium with saliva exposure. Mean ± SD + technical replicates, ANOVA

To determine adhesion strength against enzymatic detachment, the number of detached cells was measured after 2 and 6 h of cell culture using 1, 5, 10 and 20 min of serial trypsinization. Non-coated titanium with saliva exposure had significantly higher detachment levels with one minute trypsinization after 2 and 6 h of cell culture indicating weaker cell adhesion on saliva treated titanium. The difference was also significant between saliva treated titanium and saliva treated TiO_2_-coated titanium with 5 min of trypsinization. TiO_2_-coated titanium with or without saliva exposure had no significant difference in adhesion strength (Fig. 2).

### Highest cell proliferation on TiO_2_-coated titanium without saliva exposure

**Fig. 3:**
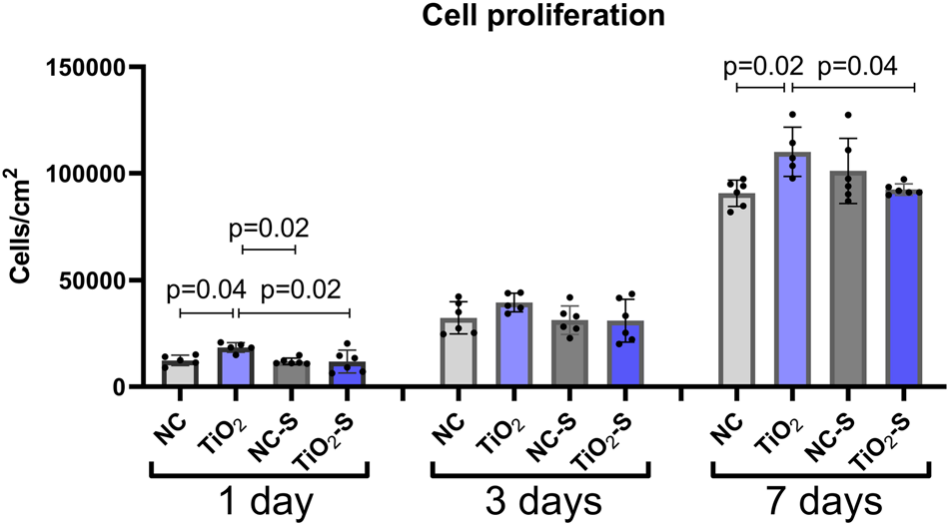
Cell proliferation levels on non-coated, TiO_2_-coated with and without saliva exposure after 1, 3 and 7 days of cell culture. NC non-coated, TiO_2_ = TiO_2_-coated, NC-S non-coated with saliva exposure, TiO_2_-S = TiO_2_-coated titanium with saliva exposure. *n* = 6 technical replicates, mean ± SD + individual values, ANOVA

To measure, whether saliva exposure affects cell growth and proliferation, HGKs were cultivated on the samples for 1, 3 and 7 days. After the first day, the proliferation level was significantly higher on TiO_2_-coated titanium compared to all other groups. Also, after one week there were significantly more cells on TiO_2_-coated titanium compared to non-coated titanium and TiO_2_-coated titanium with saliva exposure. Meanwhile the saliva treated TiO_2_-coated surface had significantly lower proliferation than the same surface before saliva treatment, the non-coated surface with saliva exposure had the opposite effect indicating higher proliferation or no effect on saliva treated titanium compared to the non-treated surface (Fig. 3).

### Saliva exposure reduces cell spreading on TiO_2_-coated surface

**Fig. 4:**
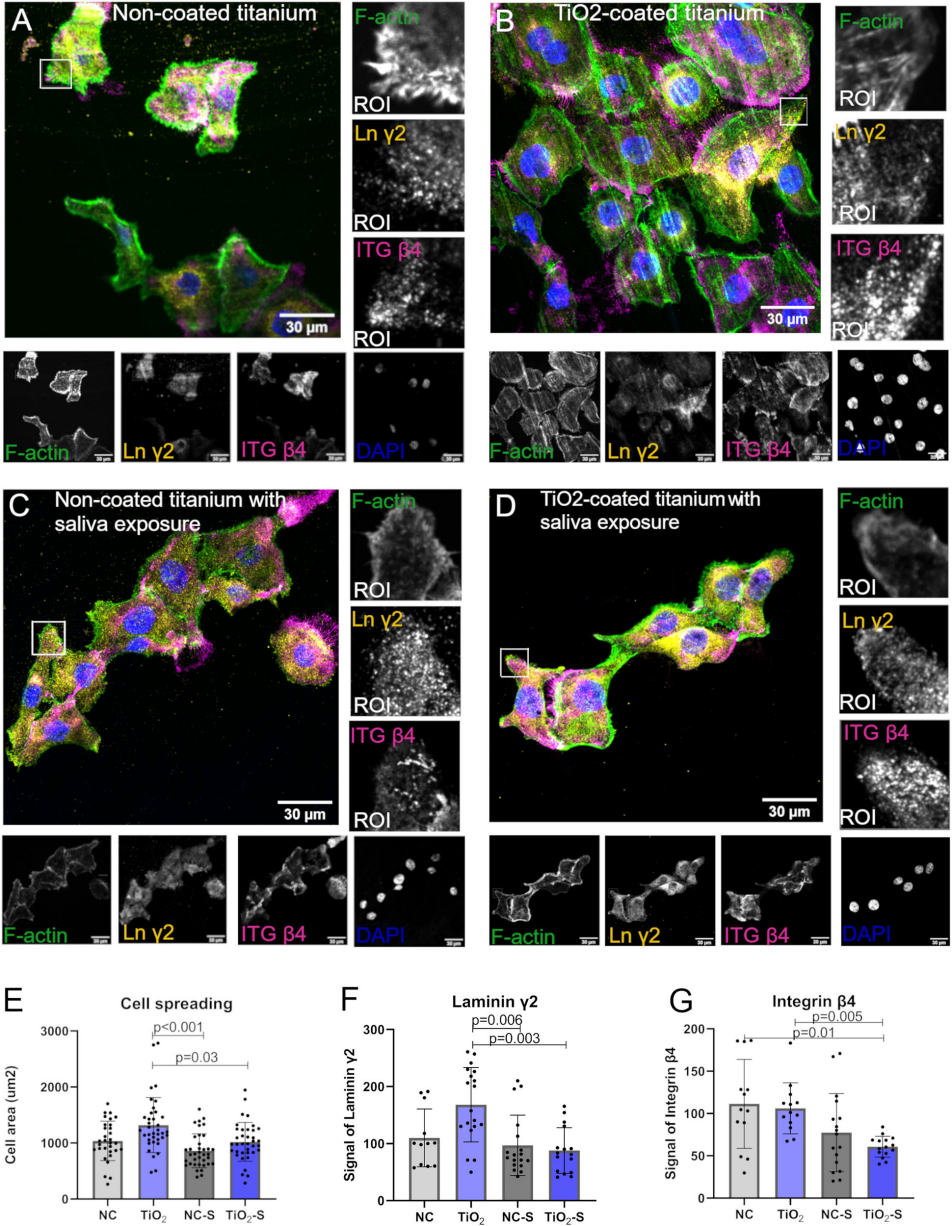
Saliva exposure reduces cell spreading and adhesion protein expression on TiO_2_-coated surface. **A**–**D** Representative confocal microscope images from the bottom plane of gingival keratinocytes stained with laminin *γ*2, integrin β4, dapi as nucleus and F-actin. Quantifications of (**E**) cell areas and signals of (**F**) Laminin *γ*2 and (**G**) Integrin β4. ROI region of interest. Mean ± SD, each data point represents a technical measurement, Kruskal Wallis test

In order to study, if results from adhesion measurements correlated with cell spreading, confocal microscope imaging of cell morphology was performed (Fig. 4). Cell spreading was analyzed based on the actin staining. More spread cells with higher density were found on TiO_2_-coated titanium. After saliva exposure, cell spreading was significantly lower on non-coated and TiO_2_-coated titanium (Fig. 4E). To study expression of Laminin-332 that binds specifically to integrin α6β4, a laminin *γ*2 subunit and Integrin β4 was stained. In line with cell spreading, signal level of Laminin *γ*2 was significantly lower on both saliva treated surfaces compared to TiO_2_-coated titanium (Fig. 4F). All the same, TiO_2_-surface after saliva exposure, had significantly lower Integrin β4 signal compared to non-coated and TiO_2_-coated titanium without saliva exposure (Fig. 4G).

## Discussion

This study evidenced higher cell proliferation, cell spreading and signals of important adhesion proteins laminin *γ*2 and integrin β4 on TiO_2_-coated titanium. The result is in the same line with earlier studies, that TiO_2_-coating produced in sol is able to enhance epithelial cell attachment and growth on titanium surface [25, 26]. Earlier studies have also revealed positive effects of sol-gel coated titanium on fibroblast and soft tissue adherence [22, 23, 28] indicating all in all enhanced cell response on bioactive TiO_2_-surface. Enhanced cell attachment on titanium surface is important, as more uniform cell adhesion makes a stronger barrier against oral microbes and consequently could decrease the risk for peri-implant infections.

However, the results evidenced that saliva exposure decreases cell attachment and growth on both surfaces and seems to neutralize the positive effects of TiO_2_-coating equalizing the cell adhesion and proliferation levels. When it comes to cell attachment to saliva treated surface, fibroblast adhesion and proliferation have been shown to be weaker to implant surface after saliva treatment [29–31]. Hirota et al. [32] also found out, that saliva contamination on commercially pure titanium decreased osteoblast growth and spreading on titanium surface. Reduced osteoblast activity was also found by Kunrath et al. [3, 7]. However, Hirota et al. [32] demonstrated, that if the discs were treated with UV after saliva contamination, the negative effects of saliva contamination were avoided. As the results indicated weaker cell attachment after saliva exposure on TiO_2_-coated titanium surface, a proper saliva control while placing implant abutment is crucial to avoid saliva contamination.

Like this study, also earlier studies have revealed induced hydrophilicity on TiO_2_-coated surface [21, 33]. In addition, this study demonstrated a decrease in WCA after saliva treatment concerning both TiO_2_-coated and non-coated titanium indicating a more hydrophilic surface after saliva exposure. Also, Schweikl et al. demonstrated lower contact angles on saliva treated titanium compared to PBS washed titanium [34]. This increase in hydrophilicity after saliva exposure can be due to adhered water molecules on the titanium surface, since saliva is mostly composed of water [5]. Hirota et al. measured WCA on saliva treated cpTi, which was around 40° [32], meanwhile our study evidenced lower than 20° WCA on non-coated saliva treated titanium. However, Kunrath et al. demonstrated loss of hydrophilic properties of titanium surface after saliva exposure [7, 35]. As WCAs have been shown to be similar on cpTi and titanium alloy after saliva treatment [36], the difference in contact angle results can be due to variations in saliva treatment methods. Besides hydrophilicity, the TiO_2_-coated surface is thought to have favorable cell response due to its nanotopography and also calcium phosphate growth on its surface [24].

In this study, no significant difference was found in total protein amounts between hydrophilic TiO_2_-surface and non-coated titanium. This is in line with previous studies, where salivary and serum protein pellicle formation on dip-coated cpTi and non-coated titanium had similar profiles [37]. Also, serum protein adsorption to nanoporous TiO_2_-coated zirconia has been tested and neither significant difference in serum protein adsorption was found [38]. According to this study, the protein adsorption seems to neutralize the bioactive effects of TiO_2_-coating and equalize the surface properties between TiO_2_-coated and non-coated surfaces.

## Conclusion

All in all, this study demonstrated lower adhesion and proliferation levels on in sol derived TiO_2_-surface after saliva exposure. Thus, a proper saliva control during the abutment placing is suggested to avoid saliva exposure of the whole abutment surface. Even though adhered saliva proteins seem to effect on cell adhesion strength and growth, clinical studies are needed to study the clinical outcome of in sol derived TiO_2_ coatings.
